# Posterior tibial slope interpretation is method‐dependent: No universal threshold for defining abnormality in primary and recurrent ACL rupture

**DOI:** 10.1002/jeo2.70808

**Published:** 2026-06-16

**Authors:** Julien Druel, Antoine Piercecchi, Clémence Peufly, Lyes Chaal, Christophe Jacquet, Romir Patel, Matthieu Ollivier

**Affiliations:** ^1^ Department of Orthopedic Surgery, Assistance Publique des Hôpitaux de Marseille, Hôpital Sainte Marguerite, St Marguerite Hospital, Institute for Locomotion Aix‐Marseille University Marseille France; ^2^ Department of Biomechanics, APHM, CNRS, ISM, St Marguerite Hospital, Institute for Locomotion Aix‐Marseille University Marseille France

**Keywords:** ACL graft failure, anterior cruciate ligament, posterior tibial slope, radiographic measurement, reference interval

## Abstract

**Purpose:**

To determine method‐specific normal posterior tibial slope (PTS) values, define abnormal PTS using two statistical approaches, and assess how abnormal PTS classification varies in primary anterior cruciate ligament (ACL) rupture and ACL re‐rupture.

**Methods:**

In this retrospective single‐centre study, all patients undergoing knee imaging during the study period were screened and categorized into three groups based on clinical and imaging findings: ACL‐intact, primary ACL rupture and ACL re‐rupture. PTS was measured on standardized lateral radiographs using five techniques. Normal values were defined as the 95% reference interval (2.5th–97.5th percentiles). Abnormal PTS was defined using both a quantile‐based approach and a standard deviation–based approach. Statistical significance was set at *p* < 0.05.

**Results:**

A total of 609 subjects were included (mean age 30 ± 5 years; 304 males and 305 females), comprising an ACL‐intact reference group (*n* = 470), a primary ACL rupture group (*n* = 88) and an ACL re‐rupture group (*n* = 51). Mean PTS in the reference group ranged from 5.9° to 8.2°, with increased PTS generally observed above 12–15°, depending on the technique. Using reference‐based thresholds, abnormal PTS was identified in 18.2%–22.7% of ACL patients and 35.3%–41.2% of ACL re‐rupture patients, with significantly higher proportions in the latter (*p* = 0.009–0.036). Using standard deviation–based thresholds, abnormal PTS was identified in 17.0%–20.5% and 27.5%–37.3% of patients, respectively. When thresholds were derived from the ACL population, abnormal PTS classification in ACL re‐rupture decreased (2.0%–9.8%) and was no longer significantly different (all *p* ≥ 0.532).

**Conclusion:**

PTS interpretation is highly method‐dependent. Normal ranges and abnormal classification vary according to measurement technique, statistical definition and reference population. A universal PTS cut‐off is therefore inappropriate.

**Level of Evidence:**

Level III, retrospective comparative cohort study.

**Abbreviations**
ACL: anterior cruciate ligament
AIR: ACL‐Intact Reference group
BMI: body mass index
CI: confidence interval
HKA: hip–knee–ankle angle
LDFA: lateral distal femoral angle
LoA: limits of agreement
MPTA: mechanical proximal tibial angle
MRI: magnetic resonance imaging
PTS: posterior tibial slope
RI: reference interval
RLCA: ACL re‐rupture
RR: relative risk
SD: standard deviation

## INTRODUCTION

Posterior tibial slope (PTS) has been widely investigated as a potential risk factor for anterior cruciate ligament (ACL) injury and graft failure [8, 28, 30]. An increased PTS has been associated with greater anterior tibial translation and altered knee biomechanics [3, 7, 21], which may help explain its proposed role in ACL injury mechanisms [12, 15, 25]. However, despite extensive investigation, the clinical utility of PTS measurements remains uncertain, and considerable debate persists regarding how to define and interpret abnormal values.

The interpretation of PTS is complicated by three fundamental methodological issues. First, multiple measurement techniques using different anatomical or mechanical reference axes yield systematically different values and variabilities [9, 29, 31, 32]. Second, existing studies typically report mean differences between groups rather than defining what constitutes a normal or abnormal value [11, 23, 28]. While statistically significant differences between injured and non‐injured populations have been reported, such comparisons do not indicate how many individuals present with extreme PTS values—a critical gap for individual risk stratification and clinical decision‐making. Third, normal PTS values have rarely been formally defined using reference intervals; abnormality is often defined using arbitrary cut‐off values [1, 26], and it remains unclear whether thresholds should be derived from healthy or ACL‐injured populations. Without method‐specific reference values and standardized definitions of abnormality, it is unclear whether reported associations between PTS and ACL injury reflect true biological relationships or methodological artefacts.

To address these limitations, normal values can be defined using the 95% reference interval (2.5th–97.5th percentiles), which is a standard approach for describing the distribution of biological variables and identifying abnormal values at the individual level, rather than for inferential comparisons between groups [10, 17]. In addition, abnormal values can be defined using mean ± 2 standard deviations (SD), a complementary method assuming an approximate normal distribution and capturing approximately 95% of observations. The use of both approaches allows a more robust and transparent characterization of extreme PTS values [2].

The primary aim of this study was to determine method‐specific normal PTS values using a 95% reference interval. The secondary aim was to evaluate how different statistical definitions of abnormality and reference populations influence the classification of abnormal PTS in primary ACL rupture and ACL re‐rupture.

It was hypothesized that (1) normal PTS values would vary according to the measurement technique, and (2) abnormal PTS classification would differ substantially depending on the statistical definition and the reference population used.

## MATERIALS AND METHODS

### Study design

This was a retrospective, single‐centre observational study conducted at a referral centre specializing in knee surgery and sports traumatology. Institutional review board approval was obtained, and the requirement for informed consent was waived (PADS22‐181). The study period was from 6 January 2025 to 18 December 2025.

### Patient identification and eligibility

All patients who underwent knee imaging during the study period, including standardized lateral knee radiographs and calibrated long‐leg radiographs, were screened for eligibility. One knee per subject was included. The index knee was defined as the clinically symptomatic side and/or the operative side.

### Exclusion criteria (applied across groups)

Prior tibial or femoral osteotomy; fractures involving the knee joint; inflammatory joint disease; advanced osteoarthritis (defined as Grade ≥ 3 according to the Kellgren–Lawrence classification) [16]; or inadequate imaging quality precluding reliable measurement of PTS or alignment parameters.

Patients aged ≥18 years were included.

Poor‐quality imaging was defined as radiographs with excessive rotational malalignment, incomplete visualization of the proximal tibia or inability to clearly identify the tibial plateau or reference axes.

### Study groups

Subjects were categorized into three groups based on clinical and imaging findings:

– *ACL‐intact reference group (AIR group, n* = *470)*: patients with meniscal lesions and MRI‐confirmed intact ACL, with no history of ligament injury or ligament reconstruction. Meniscal pathology included isolated tears without prior meniscectomy, resulting in significant meniscal deficiency.
– *Primary ACL rupture group (ACL group, n* = *88)*: patients with primary ACL rupture confirmed by clinical assessment and MRI. Concomitant meniscal tears, cartilage lesions or nonoperatively treated collateral ligament injuries were permitted.
– *ACL re‐rupture group (RLCA group, n* = *51)*: patients with ACL re‐rupture of a previously reconstructed ACL confirmed by clinical assessment and MRI evidence of graft failure. Both first‐time and multiple revision cases were included. Concomitant intra‐articular lesions and prior meniscal or cartilage procedures were recorded but not used as exclusion criteria.

MRI was required for all patients to confirm ACL status and group allocation.

**Figure 1:**
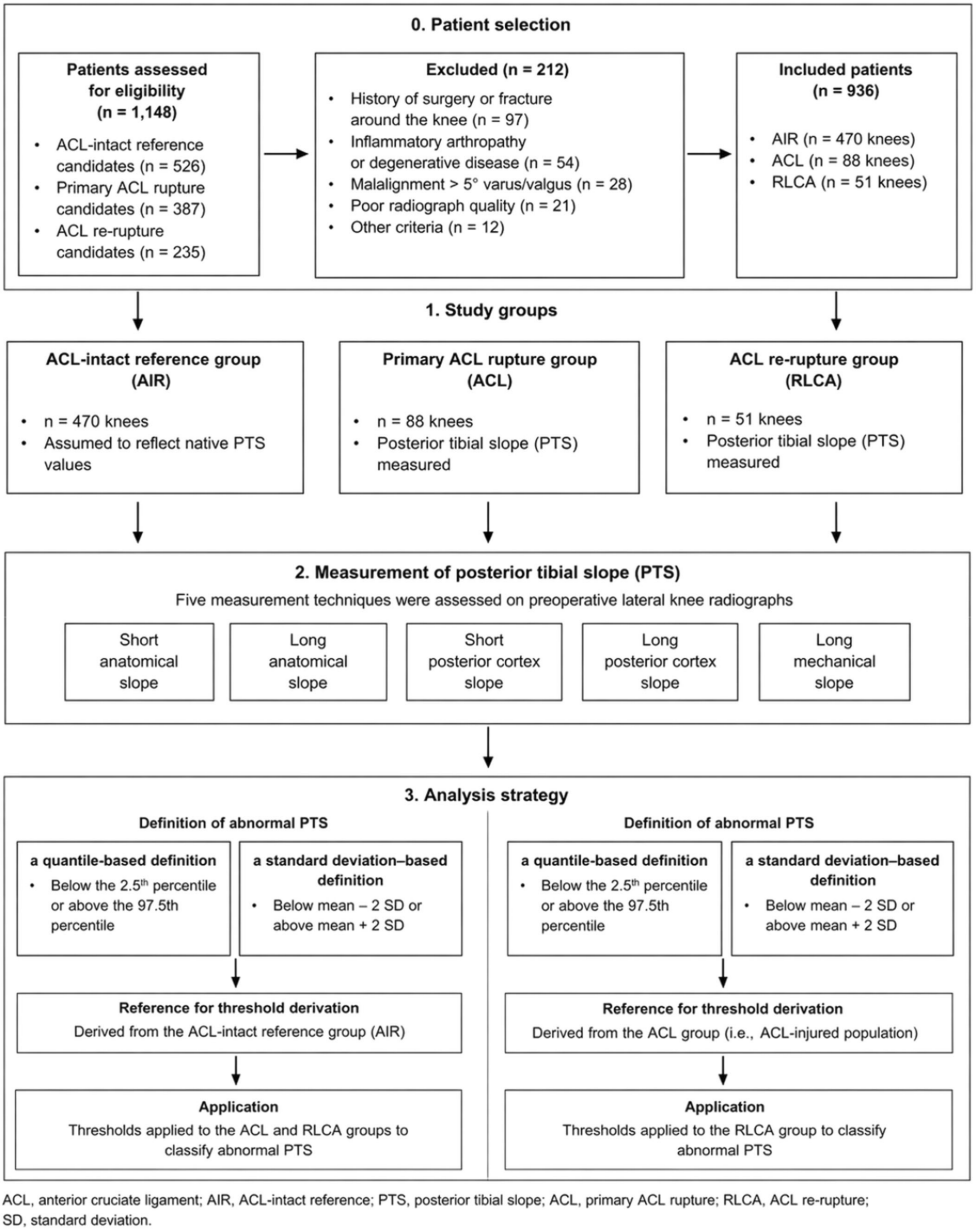
Study flowchart and analytical strategy. Patients were screened for eligibility, and exclusions were applied based on predefined criteria. The final study population was divided into an ACL‐intact reference group (AIR), a primary anterior cruciate ligament (ACL) rupture group and an ACL re‐rupture (RLCA) group. Posterior tibial slope (PTS) was measured using five techniques on lateral knee radiographs. Abnormal PTS values were defined using a quantile‐based definition (2.5th–97.5th percentiles) and a standard deviation–based definition (mean ± 2 standard deviations [SD]). Thresholds were derived from either the AIR or ACL population and applied to classify abnormal PTS.

The patient selection process is summarized in Figure 1.

### Imaging and radiographic quality criteria

All radiographs were obtained by trained radiology technicians following standardized institutional protocols.

PTS was measured on standardized lateral knee radiographs. Only true lateral views were included, defined by superimposition of the femoral condyles, which is widely accepted as a surrogate for appropriate rotational alignment. Radiographs demonstrating excessive rotational mismatch were excluded.

Acceptable rotational alignment was defined as <6 mm difference between the posterior femoral condyles, according to previously published criteria [9, 20, 31].

A minimum visible tibial length of 10–15 cm distal to the tibial plateau was required, in accordance with published recommendations to allow reliable definition of anatomical and mechanical axes [9, 20, 31].

Calibrated long‐leg radiographs were used to assess coronal alignment parameters. MRI was used to confirm ACL status and associated intra‐articular pathology.

### PTS measurement

**Figure 2:**
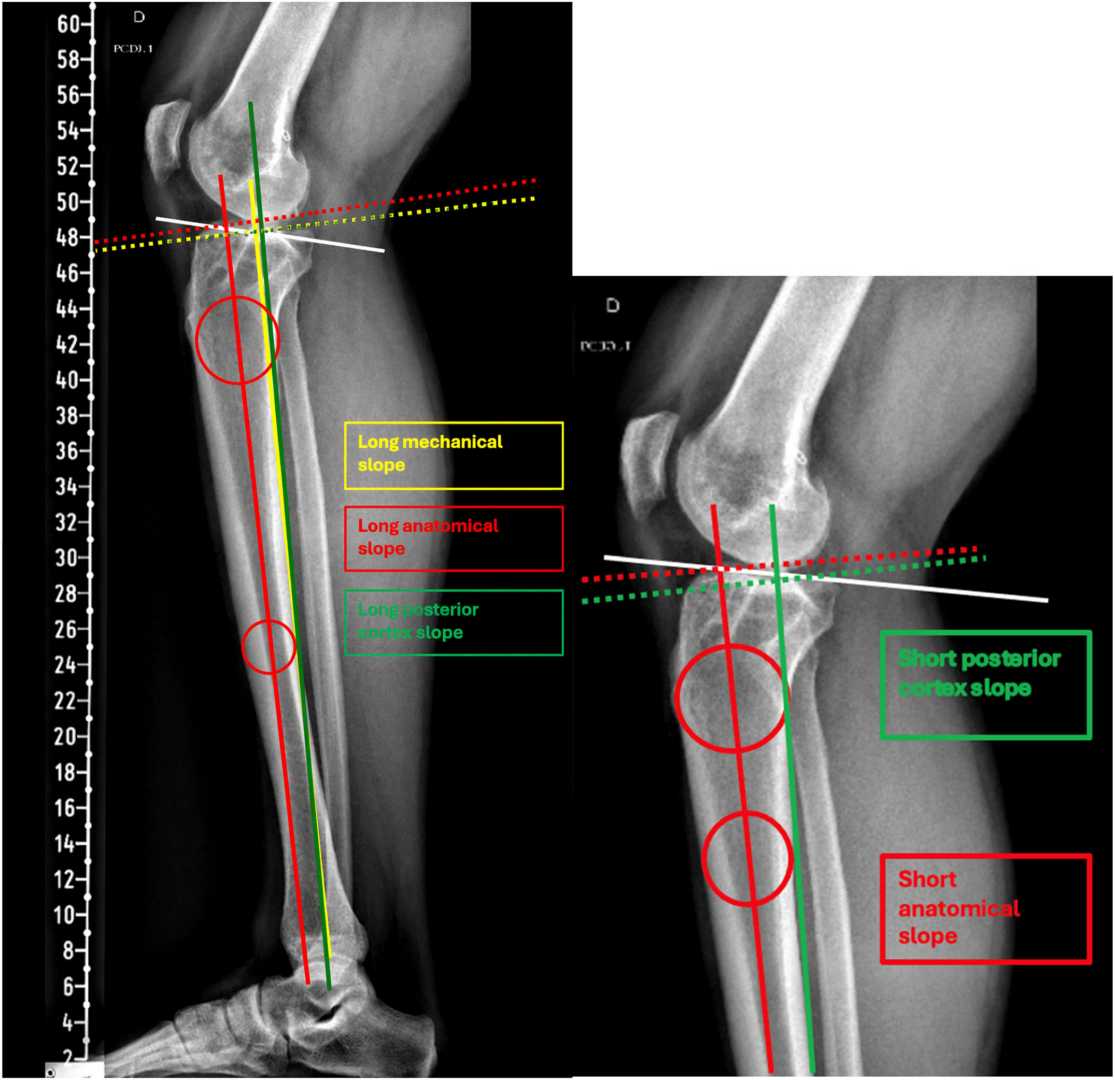
Posterior tibial slope (PTS) measurement techniques. Illustration of the five PTS measurement methods on standardized lateral knee radiographs. The PTS corresponds to the angle between a line tangent to the medial tibial plateau and a reference axis defined by the long mechanical axis of the tibia, the long anatomical axis, the short anatomical axis, and the short and long posterior tibial cortex lines. For the anatomical axis–based methods, two circular regions of interest were placed within the tibial diaphysis. For the long anatomical slope, circles were positioned approximately 5 and 10–15 cm distal to the tibial plateau, whereas for the short anatomical slope, circles were positioned approximately 5 and 7–10 cm distal to the tibial plateau. The centres of these circles defined the anatomical axis of the tibia.

PTS was measured using five techniques: short anatomical slope, long anatomical slope, short posterior cortex slope, long posterior cortex slope and long mechanical slope (Figure 2) as previously described [9, 31]. PTS was defined as the angle between a line tangent to the medial tibial plateau and the corresponding reference axis for each technique. Values were recorded using the angular convention shown in Figure 2.

For each technique, the angle between the tangent to the medial tibial plateau and the corresponding reference axis was first measured. PTS was then expressed as the complementary angle to 90°, calculated as:

PTS=90°−measured angle

All analyses, normal ranges and abnormality thresholds were calculated using this definition. Lower‐limb alignment parameters included the hip–knee–ankle angle, mechanical proximal tibial angle and lateral distal femoral angle.

### Reliability

A subset of radiographs (*n* = 50) was independently assessed by two experienced orthopaedic surgeons. Measurements were performed by two fellowship‐trained orthopaedic surgeons with specific experience in knee imaging and deformity analysis.

Measurements were repeated after a 4‐week washout period. Observers were blinded to group assignment.

Intra‐ and interobserver reliability were assessed using intraclass correlation coefficients (ICCs) calculated with a two‐way random‐effects model and absolute agreement. ICC values were interpreted according to established guidelines.

### Definitions of normal and abnormal PTS

Normal PTS values were defined separately for each measurement technique as the 95% reference interval (2.5th–97.5th percentiles), a standard approach for describing biological variability and identifying extreme values at the individual level.

Abnormal PTS values were defined using two complementary approaches:

– a quantile‐based definition (values below the 2.5th percentile or above the 97.5th percentile),
– a standard deviation–based definition (values below mean − 2 SD or above mean + 2 SD), assuming approximate normal distribution.

Thresholds were first derived from the AIR group and applied to the ACL and RLCA groups. A secondary analysis used thresholds derived from the ACL group and applied them to the RLCA group.

### Sample size calculation

An a priori sample size calculation was performed based on the expected proportion of abnormal PTS values between groups. Assumed proportions of 20% versus 40% were selected to reflect a clinically meaningful difference in abnormal PTS prevalence.

With a two‐sided alpha level of 0.05 and 80% power, the required sample size was 82 patients per group.

### Statistical analysis

Descriptive statistics were used to summarize PTS distributions for each measurement technique within the reference populations. Continuous variables are presented as mean ± SD. Normal PTS values were defined using the 95% reference interval [4, 13], and mean ± 2 SD intervals [5] were calculated as complementary thresholds.

Comparisons of continuous PTS values across groups were performed using the Kruskal–Wallis test, followed by pairwise comparisons with Holm‐adjusted *p* values.

For categorical abnormality classification, proportions of patients classified as abnormal were calculated for each technique and abnormality definition. Comparisons between ACL and RLCA groups were performed using Fisher's exact test. Relative risks (RRs) with 95% confidence intervals (CIs) were calculated to quantify effect size. Proportions are reported as percentages with 95% confidence intervals computed using the Wilson method.

Agreement analyses were performed to evaluate the consistency of abnormal PTS classification under different methodological assumptions.

First, agreement between abnormality definitions within the same reference population (reference interval vs. mean ± 2 SD) was assessed in the combined ACL and RLCA population (*n* = 139). For each measurement technique and reference population, agreement was quantified using Cohen's kappa coefficient (*κ*) [18, 24]. Directional disagreement between definitions was evaluated using McNemar's test, and the proportion of discordant classifications was calculated (Table S2A).

Second, agreement between reference populations for abnormal PTS classification was assessed within the RLCA group (*n* = 51). For each technique and abnormality definition, classifications derived from ACL‐intact reference thresholds were compared with those derived from ACL‐injured thresholds. Agreement was quantified using Cohen's kappa coefficient and McNemar's test, and discordant proportions were calculated (Table S2B).

Kappa values were interpreted according to conventional benchmarks (poor < 0.20; fair 0.21–0.40; moderate 0.41–0.60; substantial 0.61–0.80; almost perfect > 0.80) [18].

Statistical significance was set at *p* < 0.05. All analyses were performed using SPSS version 19.0 (IBM Corp.).

## RESULTS

### Study population

During the study period, a total of 856 patients were screened for eligibility. Among these, 27 patients were excluded due to advanced osteoarthritis (Kellgren–Lawrence ≥ 3), 18 due to prior osteotomy, 12 due to fractures involving the knee joint and 72 due to inadequate imaging quality (malrotation, incomplete visualization of the proximal tibia or poor delineation of the tibial plateau). The final study population consisted of 609 subjects.

The cohort included an ACL‐intact reference group (AIR, *n* = 470), a primary ACL rupture group (ACL, *n* = 88), and an ACL re‐rupture group (RLCA, *n* = 51). Baseline characteristics and lower limb alignment parameters are presented in Table 1. Groups were similar in age (*p* = 0.172), sex distribution (*p* = 0.565), and coronal alignment parameters (all *p* ≥ 0.449). Differences were observed for body mass index (*p* = 0.011) and weight (*p* = 0.008) (Table S1).

**Table 1:** Demographic characteristics and lower limb alignment.

Variable	Control group (AIR) (*n* = 470)	ACL rupture (ACL) (*n* = 88)	ACL re‐rupture (RLCA) (*n* = 51)	*p*
Age (years), mean ± SD	30.3 ± 4.7	29.6 ± 4.8	31.0 ± 5.1	0.172
Sex (male/female), n	239/231	43/45	22/29	0.565
Height (cm), mean ± SD	166.6 ± 8.9	165.1 ± 8.3	164.7 ± 8.1	0.340
Weight (kg), mean ± SD	70.7 ± 14.6	64.0 ± 13.8	67.7 ± 15.6	0.008
BMI (kg/m^2^), mean ± SD	25.4 ± 4.6	23.3 ± 4.0	24.8 ± 4.6	0.011
Kellgren–Lawrence Grade 0, *n* (%)	382 (81.3)	69 (78.4)	34 (66.7)	0.083
Kellgren–Lawrence Grade 1, *n* (%)	71 (15.1)	15 (17.0)	11 (21.6)	0.412
Kellgren–Lawrence Grade 2, *n* (%)	17 (3.6)	4 (4.5)	6 (11.7)	0.067
Concomitant meniscal lesions, *n* (%)	470 (100)	49 (55.7)	38 (74.5)	0.021
Concomitant cartilage lesions, *n* (%)	22 (4.7)	18 (20.5)	19 (37.3)	<0.001
First ACL re‐rupture/revision, *n* (%)	–	–	39 (76.5)	–
Multiple ACL re‐ruptures/revisions, *n* (%)	–	–	12 (23.5)	–
Hip–knee–ankle angle (°), mean ± SD	179.4 ± 2.7	179.8 ± 2.7	179.2 ± 3.5	0.504
Mechanical proximal tibial angle (°), mean ± SD	85.3 ± 2.4	85.4 ± 2.2	85.3 ± 2.5	0.892
Lateral distal femoral angle (°), mean ± SD	85.9 ± 2.0	85.7 ± 2.2	86.1 ± 1.9	0.449

*Note*: Values are expressed as mean ± standard deviation unless otherwise stated.

Abbreviations: ACL, anterior cruciate ligament; AIR, ACL‐Intact Reference group; BMI, body mass index; RLCA, ACL re‐rupture; SD, standard deviation.

### PTS distributions and normal values (control reference)

PTS values in the AIR group varied according to the measurement technique (Table 2). Mean PTS ranged from 5.9° to 8.2°, with standard deviations ranging from 2.6° to 3.5°.

**Table 2:** Normal posterior tibial slope values (95% reference interval) according to measurement method (control population).

Technique	Mean	SD	95% RI (2.5th–97.5th)	Mean ± 2 SD
Short anatomical slope	7.47	2.67	2.10 to 12.50	2.14 to 12.81
Long anatomical slope	8.22	3.41	1.57 to 14.53	1.41 to 15.03
Short posterior cortex slope	5.92	3.50	−0.63 to 12.55	−1.09 to 12.93
Long posterior cortex slope	6.67	2.67	1.30 to 11.70	1.34 to 12.01
Long mechanical slope	6.98	2.61	1.80 to 11.86	1.76 to 12.20

*Note*: Mean ± SD; 95% reference interval; mean ± 2 SD.

Abbreviations: RI, reference interval; SD, standard deviation.

The 95% reference intervals also varied across techniques, with upper limits ranging from approximately 11.7° to 14.5°, including slightly negative lower limits for the short posterior cortex method (Table 2). Increased PTS values were generally observed above thresholds between 12.0° and 15.0°, depending on the measurement technique.

### Group differences in PTS (continuous comparisons)

Across all five techniques, continuous PTS differed significantly among the three groups (all Kruskal–Wallis *p* ≤ 0.001, Table S1). Pairwise comparisons demonstrated higher PTS values in the ACL group compared with the AIR group, and in the RLCA group compared with the AIR group (all *p* < 0.001).

The RLCA group also showed higher PTS values than the ACL group across all techniques (Holm‐adjusted *p* ≈ 0.019–0.023, Table S1).

### Abnormal PTS classification based on AIR reference thresholds

Using controls (AIR) as the reference, abnormal PTS thresholds were defined by two approaches: quantile‐based definition (<2.5th or >97.5th percentile); SD‐based definition (mean ± 2 SD). Applying these to the ACL and RLCA groups yielded substantial variation in abnormal classification across techniques (Table 3; Table S3).

**Table 3:** Abnormal PTS using control (AIR) reference thresholds.

Definition	Technique	ACL abnormal *n*/*N* (%)	RLCA abnormal *n*/*N* (%)	*p* (RLCA vs. ACL)	RR (95% CI)
Quantile (RI)	Long anatomical slope	16/88 (18.2%)	18/51 (35.3%)	0.024	1.94 (1.09–3.46)
Quantile (RI)	Long mechanical slope	18/88 (20.5%)	21/51 (41.2%)	0.009	2.01 (1.19–3.41)
Quantile (RI)	Long posterior cortex slope	20/88 (22.7%)	21/51 (41.2%)	0.022	1.81 (1.09–3.01)
Quantile (RI)	Short anatomical slope	20/88 (22.7%)	21/51 (41.2%)	0.022	1.81 (1.09–3.01)
Quantile (RI)	Short posterior cortex slope	17/88 (19.3%)	18/51 (35.3%)	0.036	1.83 (1.04–3.22)
SD (mean ± 2 SD)	Long anatomical slope	15/88 (17.0%)	14/51 (27.5%)	0.146	1.61 (0.85–3.06)
SD (mean ± 2 SD)	Long mechanical slope	15/88 (17.0%)	19/51 (37.3%)	0.008	2.19 (1.22–3.91)
SD (mean ± 2 SD)	Long posterior cortex slope	18/88 (20.5%)	18/51 (35.3%)	0.054	1.73 (0.99–3.01)
SD (mean ± 2 SD)	Short anatomical slope	18/88 (20.5%)	18/51 (35.3%)	0.054	1.73 (0.99–3.01)
SD (mean ± 2 SD)	Short posterior cortex slope	15/88 (17.0%)	17/51 (33.3%)	0.028	1.96 (1.07–3.57)

*Note*: Counts/percent abnormal in ACL and RLCA; RLCA versus ACL inference shown as RR with 95% CI.

Abbreviations: ACL, anterior cruciate ligament; AIR, ACL‐Intact Reference group; CI, confidence interval; PTS, posterior tibial slope; RI, reference interval; RLCA, ACL re‐rupture; RR, relative risk; SD, standard deviation.

Under the quantile‐based definition, abnormal PTS was identified in 18.2%–22.7% of ACL patients and 35.3%–41.2% of RLCA patients across techniques. RLCA patients were significantly more likely than ACL patients to be classified abnormal across all techniques (*p* = 0.009–0.036), with RR ≈ 1.81–2.01 (Table 3).

**Figure 3:**
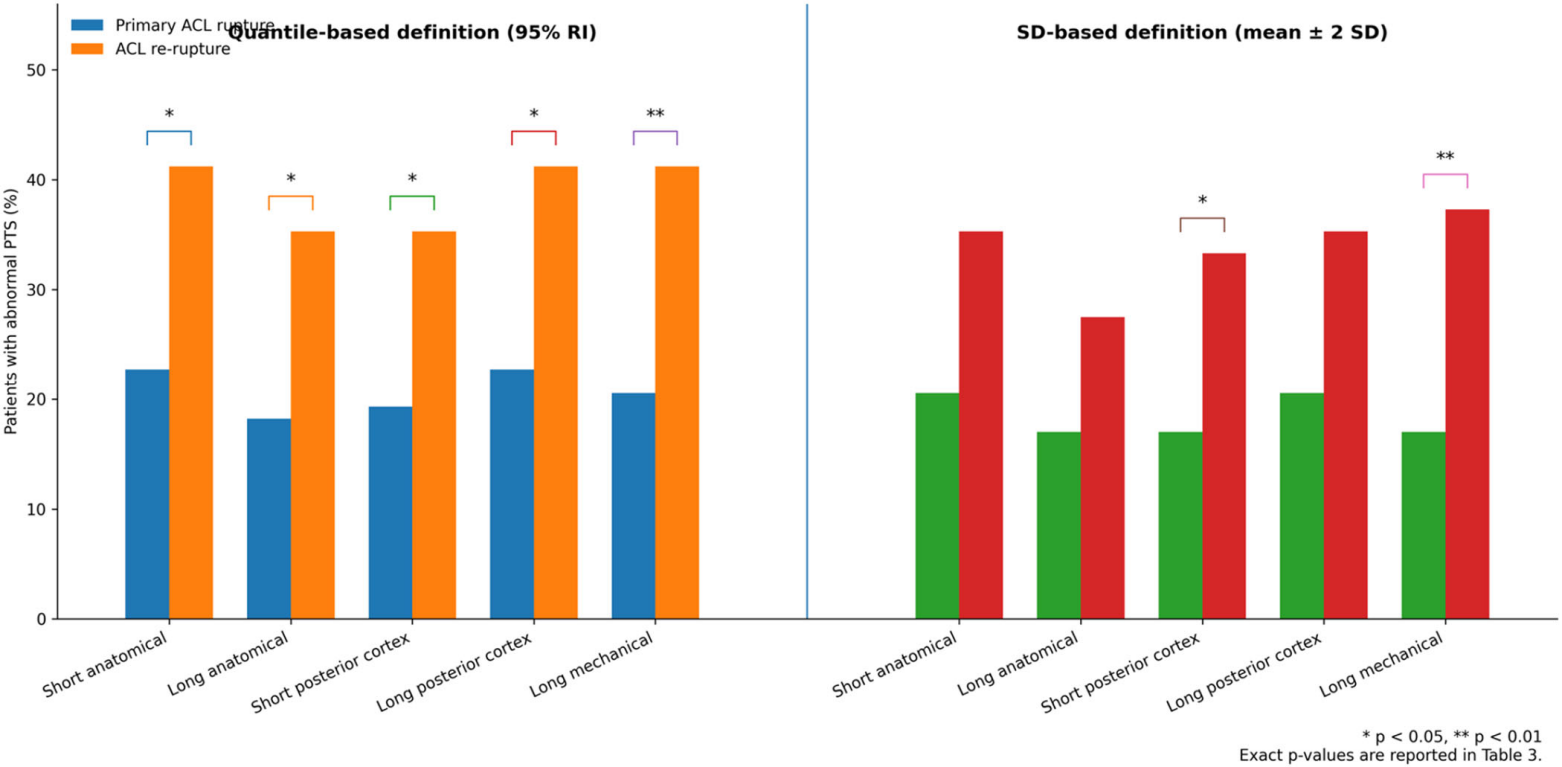
Abnormal posterior tibial slope (PTS) classification across groups. Proportion of patients with primary anterior cruciate ligament (ACL) rupture and ACL re‐rupture (RLCA) classified as having abnormal PTS values according to each measurement technique, using (A) a quantile‐based definition (2.5th–97.5th percentiles) and (B) a standard deviation–based definition (mean ± 2 standard deviations [SD]) derived from the ACL‐intact reference (AIR) population. Only statistically significant differences between groups are indicated (*p* < 0.05, *p* < 0.01). Exact *p* values are reported in Table 3. AIR, ACL‐intact reference group.

Under the SD‐based definition, abnormal PTS was identified in 17.0%–20.5% of ACL patients and 27.5%–37.3% of RLCA patients; RLCA versus ACL differences remained significant for some techniques (*p* < 0.05 for short posterior cortex and long mechanical slopes) but were not statistically significant for the remaining techniques (all *p* ≥ 0.05) (Table 3). Overall, RLCA showed a consistently higher abnormal classification rate than ACL using control‐derived thresholds (Figure 3; Table 3).

Agreement between abnormality definitions within the same reference population (reference interval vs. mean ± 2 SD) is presented in Table S2A. In the combined ACL and RLCA population (*n* = 139), agreement was high when thresholds were derived from the AIR group reference (*κ* = 0.90–0.94), with low discordance rates (2.2%–3.6%) and non‐significant McNemar tests. In contrast, when ACL‐derived thresholds were used, agreement ranged from moderate to substantial (*κ* = 0.55–0.81), with slightly higher discordance (2.2%–4.3%) and significant McNemar tests for several techniques.

### Abnormal PTS classification based on ACL‐derived thresholds

When the primary ACL group was used as the reference population, ‘normal’ ranges shifted upward across all techniques, reflecting higher PTS values in the ACL cohort (Table 4). With ACL‐based thresholds, only a small proportion of RLCA patients were classified abnormal: 7.8%–9.8% using the quantile‐based definition and 2.0%–5.9% using the SD‐based definition across techniques.

**Table 4:** Abnormal PTS using ACL reference thresholds.

Definition	Technique	ACL abnormal *n*/*N* (%)	RLCA abnormal *n*/*N* (%)	*p* (RLCA vs. ACL)	RR (95% CI)
Quantile (RI)	Long anatomical slope	6/88 (6.8%)	4/51 (7.8%)	1.000	1.15 (0.34–3.89)
Quantile (RI)	Long mechanical slope	6/88 (6.8%)	4/51 (7.8%)	1.000	1.15 (0.34–3.89)
Quantile (RI)	Long posterior cortex slope	6/88 (6.8%)	5/51 (9.8%)	0.532	1.44 (0.46–4.48)
Quantile (RI)	Short anatomical slope	6/88 (6.8%)	5/51 (9.8%)	0.532	1.44 (0.46–4.48)
Quantile (RI)	Short posterior cortex slope	6/88 (6.8%)	4/51 (7.8%)	1.000	1.15 (0.34–3.89)
SD (mean ± 2 SD)	Long anatomical slope	3/88 (3.4%)	2/51 (3.9%)	1.000	1.15 (0.20–6.66)
SD (mean ± 2 SD)	Long mechanical slope	4/88 (4.5%)	3/51 (5.9%)	0.707	1.29 (0.30–5.55)
SD (mean ± 2 SD)	Long posterior cortex slope	3/88 (3.4%)	2/51 (3.9%)	1.000	1.15 (0.20–6.66)
SD (mean ± 2 SD)	Short anatomical slope	3/88 (3.4%)	2/51 (3.9%)	1.000	1.15 (0.20–6.66)
SD (mean ± 2 SD)	Short posterior cortex slope	3/88 (3.4%)	1/51 (2.0%)	1.000	0.58 (0.06–5.39)

*Note*: Counts/percent abnormal in ACL and RLCA; RLCA versus ACL inference shown as RR with 95% CI.

Abbreviations: ACL, anterior cruciate ligament; AIR, ACL‐Intact Reference group; CI, confidence interval; PTS, posterior tibial slope; RI, reference interval; RLCA, ACL re‐rupture; RR, relative risk; SD, standard deviation.

**Figure 4:**
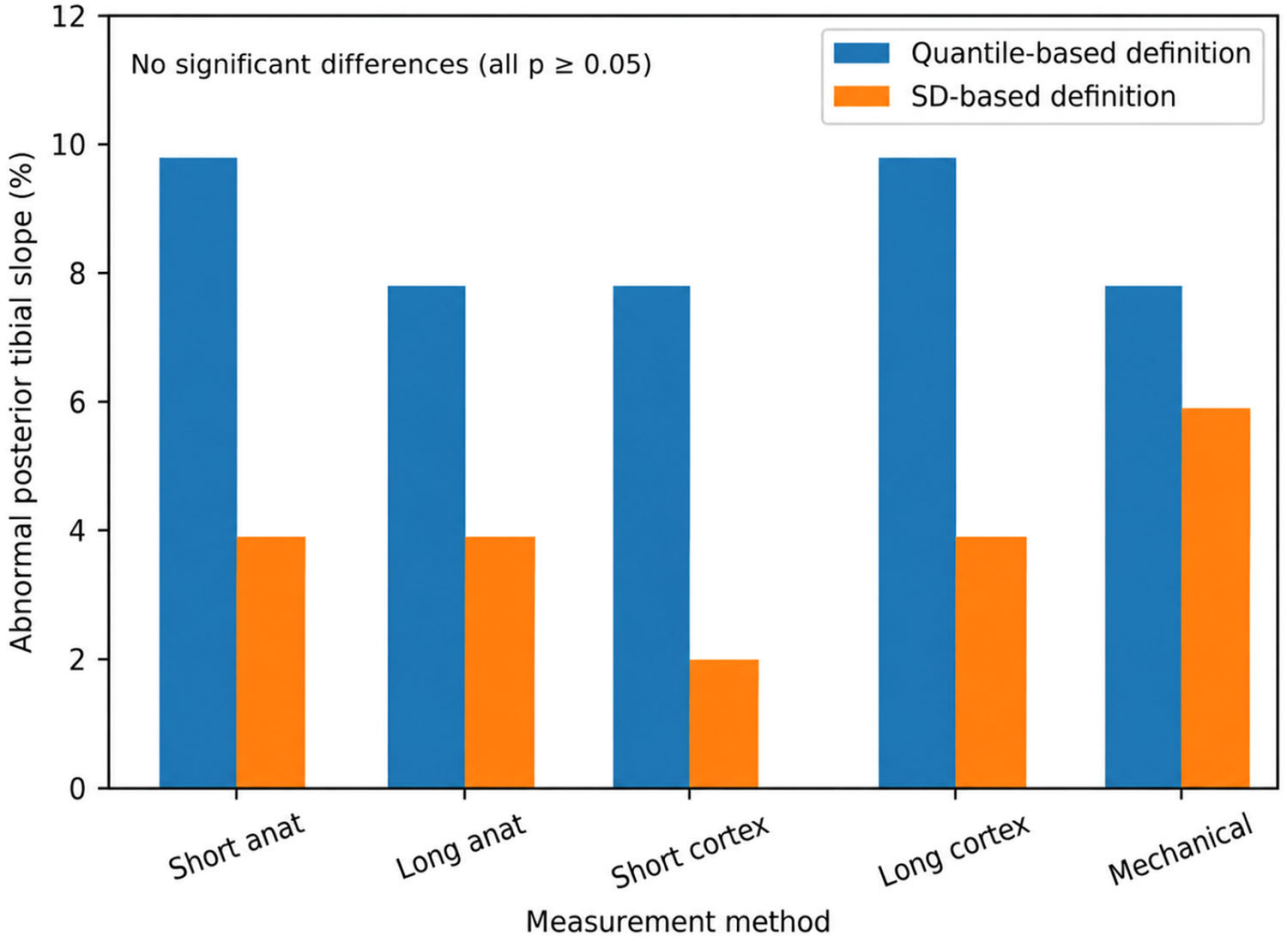
Abnormal posterior tibial slope (PTS) classification using anterior cruciate ligament (ACL)‐derived thresholds. Proportion of patients with ACL re‐rupture (RLCA) classified as having abnormal PTS values according to each measurement technique, using (A) a quantile‐based definition (2.5th–97.5th percentiles) and (B) a standard deviation–based definition (mean ± 2 standard deviations [SD]) derived from the ACL population.

Under these ACL‐derived definitions, RLCA abnormality rates were not significantly different from ACL across all techniques (all *p* ≥ 0.532, Table 4), indicating substantial dependence of ‘abnormal’ classification on the reference population (Figure 4; Table 4).

### Agreement between reference populations

Agreement between thresholds derived from the AIR and ACL populations was low in the RLCA group (*κ* = 0.03–0.27), with high discordance rates (23.5%–41.2%) and significant McNemar tests (all *p* < 0.001), indicating substantial differences in abnormal classification depending on the reference population used (Table S2B).

### Reliability

Intraobserver and interobserver reliability were high across all measurement techniques. ICCs ranged from 0.85 to 0.94 for intraobserver reliability and from 0.82 to 0.90 for interobserver reliability, indicating good to excellent agreement.

## DISCUSSION

This study showed that abnormal PTS classification varied substantially depending on the measurement technique, the statistical definition of abnormality, and the reference population used. Differences in abnormal PTS prevalence were observed between primary ACL rupture and ACL re‐rupture, particularly when thresholds were derived from the ACL‐intact reference population. These findings suggest that PTS interpretation is highly method dependent and may explain inconsistencies reported in the literature.

A central implication of this study is that group‐level comparisons do not adequately address the clinical question of how frequently individuals present with extreme values. In this study, abnormal PTS values were defined as values falling outside the 95% reference interval (i.e., below the 2.5th percentile or above the 97.5th percentile) or beyond mean ± 2 SDs. While previous studies have reported mean differences in PTS between injured and non‐injured populations, such differences do not provide information on the proportion of patients with values at the extremes of the distribution [6, 8, 19, 28]. The present findings therefore support an approach based on individual‐level classification rather than group‐level comparisons alone.

The observed variation in reference ranges across measurement techniques highlights that technique choice influences both central tendency and dispersion. This is consistent with prior reports that the tibial reference axis materially affects PTS values and their variability [22, 27]. As a result, applying a single cut‐off value across different measurement techniques is not appropriate. Instead, thresholds for increased PTS should be interpreted within the context of the specific measurement method used. Although thresholds for increased PTS were generally observed between approximately 12° and 15°, these values were method dependent and should not be considered universally applicable.

The presence of slightly negative values within the reference interval for certain techniques highlights that statistical definitions of normality may include values that do not strictly reflect anatomical posterior slope but rather measurement variability. These values were close to zero and located in the extreme lower tail of the distribution and are unlikely to be clinically relevant. This observation reinforces that PTS interpretation should primarily focus on increased values rather than lower extremes.

When thresholds were derived from the control reference population, abnormal PTS values were common in both ACL rupture and ACL re‐rupture, and consistently more prevalent in ACL re‐rupture than primary rupture. Using quantile‐based abnormality, approximately 18%–23% of ACL‐injured patients and 35%–41% of ACL re‐rupture patients were classified as outliers, with slightly lower values using the standard deviation–based definition. This pattern suggests that abnormal PTS values are enriched in the ACL re‐rupture setting, but it also emphasizes an important nuance: the magnitude of enrichment varies by measurement technique, meaning the apparent strength of association between PTS and the clinical phenotype is method dependent.

The most instructive finding was the marked reduction in ‘abnormality’ when thresholds were derived from the ACL‐injured population. Under ACL‐derived cut‐points, only a small minority of ACL re‐rupture patients remained outliers (8%–10% using quantiles and 2%–6% using standard deviation criteria). This should be interpreted as a methodological consequence of redefining normality relative to an already injured distribution. If the reference distribution is shifted and/or broadened in ACL‐injured knees, then fewer ACL re‐rupture knees will meet outlier criteria by design. In other words, abnormality becomes a *relative* label anchored to the chosen reference population, and discriminating within the injury spectrum becomes inherently more difficult.

These results provide a practical explanation for heterogeneity in the literature on PTS and ACL injury [6, 14, 21]. Apparent disagreements between studies may arise from differences in measurement technique, reference population and abnormality definition rather than true biological contradictions [1, 25]. Without explicit method‐specific reporting and transparent threshold derivation, it is difficult to interpret why one study identifies a strong association while another does not. A consistent interpretive framework—anchored to technique‐specific reference intervals—may improve comparability and reduce misleading cross‐study inferences.

From a clinical perspective, these results suggest that PTS should not be interpreted using a universal cut‐off value. Instead, PTS should be evaluated using method‐specific reference intervals, with clear reporting of how thresholds are derived. Furthermore, increased PTS values should be considered as one component of a multifactorial risk profile rather than an isolated determinant of clinical decision‐making. Future research should focus on integrating PTS into comprehensive risk models that include additional anatomical, biomechanical, and clinical factors.

Several limitations should be acknowledged. This was a retrospective single‐centre study, which may limit generalizability. The reference population comprised patients with meniscal lesions and MRI‐confirmed intact ACL and may not fully represent an asymptomatic cohort. PTS was measured on two‐dimensional radiographs and does not account for three‐dimensional morphology. Finally, the number of ACL re‐rupture cases was limited, reducing precision and potentially amplifying technique‐dependent variability. In addition, the ACL re‐rupture group was heterogeneous and included both first‐time and multiple revision cases, which may have influenced PTS distributions and abnormal classification rates. Furthermore, the present study evaluated radiographic findings at a single time point and did not assess longitudinal clinical outcomes or graft survival over time.

## CONCLUSION

Interpretation of PTS was found to be highly method dependent. Normal ranges and abnormal classification varied substantially according to measurement technique, statistical definition and reference population. These findings indicate that a single universal PTS cut‐off is not appropriate, and that PTS should be interpreted using method‐specific reference intervals with transparent reporting of threshold derivation.

The AIR group was used to derive abnormality thresholds; therefore, abnormal proportions are not reported for this group.

## AUTHOR CONTRIBUTIONS

Julien Druel contributed to study conception and design, data collection, radiographic measurements, statistical analysis and manuscript drafting. Antoine Piercecchi, Clémence Peufly and Lyes Chaal contributed to data collection, radiographic measurements and manuscript revision. Christophe Jacquet and Romir Patel contributed to data interpretation, methodological supervision and critical revision of the manuscript. Matthieu Ollivier supervised the study, contributed to study design, data interpretation and critical revision of the manuscript.

## FUNDING INFORMATION

The authors have no funding to report.

## CONFLICT OF INTEREST STATEMENT

The authors declare no conflicts of interest.

## ETHICS STATEMENT

This study was approved by the local Institutional Review Board (Comité d’éthique de l'Institut du Mouvement et de l'Appareil Locomoteur, Aix‐Marseille Université) under approval number IRB‐2023‐ACL‐07.

## Supporting information


**Table S1.** Continuous PTS comparisons across groups (overall + pairwise Holm‐adjusted p values).

**Table S2A.** Agreement between abnormality definitions within the same reference population (RI vs mean±2 SD). Population: ACL + RLCA combined (n = 139). Interpretation: High agreement under AIR reference, moderate–substantial under ACL reference.

**Table S2B.** Agreement between reference populations for RLCA classification (AIR‐threshold vs ACL‐threshold). Population: RLCA only (n = 51). Interpretation: Very low kappa + high discordance; McNemar highly significant across all techniques/definitions → strong directional disagreement.

**Table S3.** Bland–Altman summary (bias and limits of agreement).

## Data Availability

The data that support the findings of this study are available from the corresponding author upon reasonable request. Due to institutional and ethical restrictions, the data are not publicly available.
