# Supplementary material for: Posterior tibial slope interpretation is method‐dependent: No universal threshold for defining abnormality in primary and recurrent ACL rupture
Source: J Exp Orthop. 2026 Jun 16;13(2):e70808. doi: 10.1002/jeo2.70808 (PMC13270395; doi:10.1002/jeo2.70808)
Supplement: Supplementary file 1 — Table S1. Continuous PTS comparisons across groups (overall + pairwise Holm‐adjusted p values). [file JEO2-13-e70808-s001.docx]

**Supplementary Table S1. Continuous PTS comparisons across groups (overall + pairwise Holm-adjusted p values)**

| **Technique** | **AIR (n=470) Mean ± SD** | **95% CI** | **ACL (n=88) Mean ± SD** | **95% CI** | **RLCA (n=51) Mean ± SD** | **95% CI** | **Overall p** | **ACL vs AIR** | **RLCA vs AIR** | **RLCA vs ACL** |
| --- | --- | --- | --- | --- | --- | --- | --- | --- | --- | --- |
| Short anatomical slope | 7.5 ± 2.7 | 7.2–7.7 | 9.5 ± 2.7 | 8.9–10.1 | 10.5 ± 2.7 | 9.7–11.3 | p < 0.001 | p < 0.001 | p < 0.001 | 0.022 |
| Long anatomical slope | 8.2 ± 3.4 | 7.9–8.5 | 10.2 ± 3.4 | 9.4–11.0 | 11.3 ± 3.4 | 10.4–12.2 | p < 0.001 | p < 0.001 | p < 0.001 | 0.019 |
| Short posterior cortex slope | 5.9 ± 3.5 | 5.6–6.2 | 7.9 ± 3.5 | 7.1–8.7 | 8.9 ± 3.5 | 7.9–9.9 | p < 0.001 | p < 0.001 | p < 0.001 | 0.021 |
| Long posterior cortex slope | 6.7 ± 2.7 | 6.4–6.9 | 8.7 ± 2.7 | 8.1–9.3 | 9.7 ± 2.7 | 8.9–10.5 | p < 0.001 | p < 0.001 | p < 0.001 | 0.022 |
| Long mechanical slope | 7.0 ± 2.6 | 6.7–7.2 | 9.0 ± 2.6 | 8.4–9.6 | 10.0 ± 2.6 | 9.2–10.8 | p < 0.001 | p < 0.001 | p < 0.001 | 0.023 |
