# Supplementary material for: Posterior tibial slope interpretation is method‐dependent: No universal threshold for defining abnormality in primary and recurrent ACL rupture
Source: J Exp Orthop. 2026 Jun 16;13(2):e70808. doi: 10.1002/jeo2.70808 (PMC13270395; doi:10.1002/jeo2.70808)
Supplement: Supplementary file 2 — Table S2A. Agreement between abnormality definitions within the same reference population (RI vs mean±2 SD). Population: ACL + RLCA combined (n = 139). Interpretation: High agreement under AIR reference, moderate–substantial under ACL reference. [file JEO2-13-e70808-s002.docx]

**Supplementary Table S2A. Agreement between abnormality definitions within the same reference population (RI vs mean±2SD)**

Population: ACL + RLCA combined (n = 139)

Interpretation: High agreement under AIR reference, moderate–substantial under ACL reference.

| **Reference population** | **Technique** | **Kappa** | **McNemar p** | **Discordant (%)** | **n** |
| --- | --- | --- | --- | --- | --- |
| **AIR** | Short anatomical slope | 0.91 | 0.074 | 3.6 | 139 |
| **AIR** | Long anatomical slope | 0.90 | 0.074 | 3.6 | 139 |
| **AIR** | Short posterior cortex slope | 0.94 | 0.248 | 2.2 | 139 |
| **AIR** | Long posterior cortex slope | 0.91 | 0.074 | 3.6 | 139 |
| **AIR** | Long mechanical slope | 0.91 | 0.074 | 3.6 | 139 |
| **ACL** | Short anatomical slope | 0.61 | 0.041 | 4.3 | 139 |
| **ACL** | Long anatomical slope | 0.65 | 0.074 | 3.6 | 139 |
| **ACL** | Short posterior cortex slope | 0.55 | 0.041 | 4.3 | 139 |
| **ACL** | Long posterior cortex slope | 0.61 | 0.041 | 4.3 | 139 |
| **ACL** | Long mechanical slope | 0.81 | 0.248 | 2.2 | 139 |
