# Supplementary material for: Posterior tibial slope interpretation is method‐dependent: No universal threshold for defining abnormality in primary and recurrent ACL rupture
Source: J Exp Orthop. 2026 Jun 16;13(2):e70808. doi: 10.1002/jeo2.70808 (PMC13270395; doi:10.1002/jeo2.70808)
Supplement: Supplementary file 3 — Table S2B. Agreement between reference populations for RLCA classification (AIR‐threshold vs ACL‐threshold). Population: RLCA only (n = 51). Interpretation: Very low kappa + high discordance; McNemar highly significant across all techniques/definitions → strong directional disagreement. [file JEO2-13-e70808-s003.docx]

**Supplementary Table S2B. Agreement between reference populations for RLCA classification (AIR-threshold vs ACL-threshold)**

Population: RLCA only (n = 51)

Interpretation: Very low kappa + high discordance; McNemar highly significant across all techniques/definitions → strong directional disagreement.

| **Definition** | **Technique** | **Kappa** | **McNemar p** | **Discordant (%)** | **n** |
| --- | --- | --- | --- | --- | --- |
| **Quantile (RI)** | Short anatomical slope | 0.09 | 0.000796 | 39.2 | 51 |
| **Quantile (RI)** | Long anatomical slope | 0.27 | 0.000512 | 27.5 | 51 |
| **Quantile (RI)** | Short posterior cortex slope | 0.27 | 0.000512 | 27.5 | 51 |
| **Quantile (RI)** | Long posterior cortex slope | 0.09 | 0.000796 | 39.2 | 51 |
| **Quantile (RI)** | Long mechanical slope | 0.03 | 0.000480 | 41.2 | 51 |
| **Mean ± 2SD** | Short anatomical slope | 0.03 | 0.000407 | 35.3 | 51 |
| **Mean ± 2SD** | Long anatomical slope | 0.19 | 0.001 | 23.5 | 51 |
| **Mean ± 2SD** | Short posterior cortex slope | 0.08 | 0.000177 | 31.4 | 51 |
| **Mean ± 2SD** | Long posterior cortex slope | 0.03 | 0.000407 | 35.3 | 51 |
| **Mean ± 2SD** | Long mechanical slope | 0.09 | 0.000407 | 35.3 | 51 |
