# Supplementary material for: Posterior tibial slope interpretation is method‐dependent: No universal threshold for defining abnormality in primary and recurrent ACL rupture
Source: J Exp Orthop. 2026 Jun 16;13(2):e70808. doi: 10.1002/jeo2.70808 (PMC13270395; doi:10.1002/jeo2.70808)
Supplement: Supplementary file 4 — Table S3. Bland–Altman summary (bias and limits of agreement). [file JEO2-13-e70808-s004.docx]

**Supplementary Table S3. Bland–Altman summary (bias and limits of agreement)**

| **Pair** | **Bias (°)** | **LoA low (°)** | **LoA high (°)** |
| --- | --- | --- | --- |
| Short anatomical vs Long anatomical | −0.81 | −4.59 | 2.97 |
| Long anatomical vs Long mechanical | 1.32 | −2.53 | 5.16 |
| Long posterior cortex vs Long mechanical | −0.29 | −1.24 | 0.66 |
| Short posterior cortex vs Long posterior cortex | −0.68 | −4.53 | 3.16 |
